# The Effect of STAT3 Signal Pathway Activation on Retinopathy of Prematurity

**DOI:** 10.3389/fped.2021.638432

**Published:** 2021-11-10

**Authors:** Jianbing Ren, Jingbo Jiang, Weiming Ou, Xianqiong Luo, Jianwen Xiang, Guosheng Liu, Shuiqing Huang, Longkai He, Jiamin Gan, Hongping Li, Chuan Nie

**Affiliations:** ^1^Department of Neonatology, Guangdong Women and Children Hospital, Guangzhou, China; ^2^Department of Neonatology, Shenzhen Children's Hospital, Shenzhen, China; ^3^Department of Neonatology and Pediatrics, The First Affiliated Hospital, Jinan University, Guangzhou, China

**Keywords:** retinopathy of prematurity, STAT3, vascular endothelial growth factor, HAMP, STAT3 signal pathway

## Abstract

**Objective:** To investigate the mechanism of activation of the signal transducer and activator of transcription 3 (STAT3) signal pathway in the process of retinopathy of prematurity (ROP).

**Methods:** Sixty newborn Sprague-Dawley (SD) rats were randomly separated into the hyperoxia and air control groups (*n* = 30/in each group). The serum hepcidin level on 21 d was measured using the enzyme-linked immunosorbent assay (ELISA). The expression of HAMP and STAT3 protein in the liver was determined using reverse transcription-polymerase chain reaction (RT-PCR) and western blotting. Retinal neovasculature was evaluated by hematoxylin and eosin (HE) stain and fluorescein lectin. The retinal endothelial cells were treated with 250 μmol/L cobalt chloride for 72 h and added S3I-201. The STAT3 level was determined by western blotting.

**Results:** The expression of STAT3 protein increased significantly after hyperoxia stimulation. The expression of HAMP mRNA in the hyperoxia group was significantly higher than that of the control group. The proliferation of retinal cells was inhibited, and the expression of STAT3 was increased. No significant difference was noted in vascular endothelial growth factor (VEGF) mRNA. The expression of STAT3 and VEGF mRNA was significantly reduced.

**Conclusion:** The activation of the STAT3 signal pathway increased hepcidin expression, contributing to the pathogenesis of ROP. S3I-201 inhibited the expression of STAT3 and VEGF mRNA levels. This information provides potential novel therapeutic approach to the prevention and treatment of ROP.

## Introduction

Retinopathy of prematurity (ROP) is vascular proliferative disease of the retina, mainly affecting premature infants with low birth weight (1, 2). Approximately 15 million premature babies are born globally every year, which accounts for 1/10 of newborns. About 1.2 million premature babies are born every year in China, ranking it as the second globally. The multicenter survey of 2015 on retinopathy of preterm infants indicated a 15.2% ROP incidence on the Chinese mainland. The incidence in premature infants under gestational age of 30 weeks was 30.6% (3). ROP is the leading cause of childhood blindness disease in developed countries and some developing countries (4–6).

The main pathological features of ROP are including abnormal angiogenesis, increased vascular leakage, vascular tortuosity, and dilatation. These features result in serious ocular complications and even blindness. The clear pathogenesis of ROP primarily includes an abnormal signal pathway of vascular endothelial growth factor (VEGF), which leads to abnormality in its secretion and proliferation of retinal vessels. Exogenous factors such as oxidative stress, inflammation, nutritional factors, hypoxia-inducible factor (HIF), and neuroprotective growth factor promote ROP occurrence (6, 7). Currently, non-standard oxygen use, low gestational age, and low birth weight have been identified as major risk factors causing ROP (8, 9). Although some studies have revealed that some drugs such as anti-vascular endothelial growth factor and antioxidants can control ROP progression; however, the administration timing is unknown (10, 11). Subsequently, a meta-analysis of Fang JL et al. indicated a lack of high-quality evidence that effective drug interventions can prevent the ROP occurrence (12). Nonetheless, oxygen administration and retinal immaturity leads to retinal vascular hyperplasia. Therefore, screening, early diagnosis, and treatment through photocoagulation or vitreous injection of bevacizumab could be used in ROP (13).

Janus tyrosine kinase (JAK) /signal transducer and activator of transcription (STAT) signal pathway is a signal transduction pathway closely related to neovascularization. STAT is phosphorylated by JAK kinases in response to cytokine activation such as growth hormone and IL-6 family cytokines. Its transmission process is relatively simple compared to other signal pathways, mainly composed of three components such as JAK, tyrosine kinase-related receptor, and STAT (14, 15). It is crucial to specifically target pathological neovascularization rather than targeting normal blood vessels when proliferative retinal diseases are taken into consideration, such as malignant tumors.

VEGF is an important cytokine of retinal vascular proliferation in ROP. Hyperoxia can increase the production of reactive oxygen species (ROS), nitric oxide synthase (NOS), expression of inflammatory cytokines and VEGF, and retinal angiogenesis, but its specific regulatory mechanism is uncertain. Researchers use STAT3 antisense oligodeoxynucleotides and dominant-negative STAT3 proteins to block STAT3 signal transduction. These can down-regulate VEGF expression, suggesting that STAT3 can bind to the VEGF promoter and directly regulate its gene (16–19). If there is a mutation in the STAT3 binding site in the VEGF promoter, it can cause the loss of the VEGF promoter activity and subsequently inhibit VEGF expression (18, 20).

In addition, iron transport balance and other pathways may perhaps play a key role in the occurrence and development of ROP. Blood transfusion and increased iron load are the high-risk factors of ROP. Hepcidin (encoded by HAMP) is the Fe-dependent inhibitor protein of rat liver microsomal 3-hydroxy-3-methylglutaryl-CoA (HMGCoA) reductase, which interacts with Fe (21). HMGCoA reductase activity was inhibited by the addition of FeSO_4_ and the cytosolic protein hepcidin (22). Occasionally, hepcidin is the key factor that regulates iron balance, and the JAK/STAT3 signal pathway regulates its expression.

Taken together, we hypothesized that hyperoxia could activate the STAT3 pathway, stimulate the expression of the key factor of iron balance, and then promote VEGF expression. STAT3 pathway inhibitor S3I-201 can block this effect and prevent ROP. In this study, we intended to outline the effects of hyperoxia on the STAT3 signal pathway activation, hepcidin, and VEGF expression, which aimed to further enrich the pathogenesis of ROP by providing a new way for its prevention and treatment.

## Materials and Methods

### Ethical Consideration

The study was conducted in accordance with Guangdong Medical Laboratory Animal Center, PR China (SCXK 2013-0002) and approved by the ethics committee of Guangdong Women and Children Hospital (#201701081).

### Specimen Collection

Sixty newborn Sprague-Dawley (SD) rats from Guangdong Medical Laboratory Animal Center were randomly divided into the ROP model group (*n* = 30) and control group (*n* = 30). The ROP model group was fed alternately with high oxygen concentration (50% ±2) and low oxygen concentration (10% ±2) every day and returned to the air 14 days after birth. The air group was kept in the air all the time, and both groups were fed until 20 days. On the 21 days after birth, the rats were anesthetized with 10% chloral hydrate. After decapitation, the blood samples were incubated at room temperature for 30 min. The blood samples were centrifuged at the speed of 3,600 r/min in a centrifuge. The supernatant was stored in −20°C refrigerator for further use. The right lobe of the liver was cryopreserved in an ultra-low temperature refrigerator at −80°C. The number of retinal vessels was observed through hematoxylin and eosin (HE) staining, and the retina of rats were obtained under an anatomical microscope. The retinal vessels were labeled with fluorescein lectin to comprehend the ischemic retinal area, vascular branches, and the neovascularization plexus.

### Quantitative Real-Time Polymerase Chain Reaction

Using Trizol reagent (#15596-026, Life Technologies, USA) to extract mRNA, DNA reverse transcription (#K1622, Thermo Scientific, USA) from liver tissue, according to the kit's instructions, primers were designed and synthesized based on the target gene sequence (Table 1). RT-PCR measured the target gene expression under the following conditions: 35 cycles, 92°C 30 s, 58°C 45 s, and 72°C 35 s. The data were collected, and all the typical CT values were calculated. The samples based on quantitative fluorescence analysis were drawn with GAPDH as the internal reference. The standard curve was drawn for semi-quantitative analysis.

**Table 1:** Primer sequences were used for qPCR in this study.

**Name**	**Forward primer (5^′^−3^**′**^)**	**Reverse primer (5^**′**^– 3^**′**^)**
hVEGF	AAGGAGGAGGGCAGAAT	AAGATGTCCACCAGGGTC
rHAMP	CTGCCTGTCTCCTGCTTC	TTGGTGTCTCGCTTCCTT
hGAPDH	CAGGAGGCATTGCTGATGAT	GAAGGCTGGGGCTCATTT
rGAPDH	GACATGCCGCCTGGAGAAAC	AGCCCAGGATGCCCTTTAGT

### Enzyme-Linked Immunosorbent Assay

According to the manufacturer's instructions, the levels of hepcidin in the serum were detected using an enzyme-linked immunosorbent assay kit (#EK0412, Boster Biological Technology, China). Briefly, 50 μl enzyme-labeled reagents were added to the 96-well-plate, except the blank control. After mixing, the orifice plates were incubated at 37°C for 30 min, followed by the addition of reaction substrates A and B (50 μl each time), and then dark incubation at 37°C for 10 min. The absorbance value of 450 nm wavelength was measured by a microplate reader (ThermoFisher, USA) after adding quenching solution for 15 min. The regression method is used to study the optical density (OD) value to deduce the sample concentration further.

### Western Blotting

The IL-6R, STAT3 protein expressed levels in the liver were detected by western blot. Proteins were extracted with RIPA lysis buffer (#P0013B, Beyotime, China). Protein content was determined by Coomassie brilliant blue method and stored in a 20°C refrigerator for further tests. The protein was then separated by 10% sodium dodecyl sulfate-polyacrylamide gel electrophoresis (SDS-PAGE), and the isolated protein was transferred into polyvinylidene fluoride (PVDF) membrane by semi-dry method (100 mA 1.5 h). At room temperature, 5% skimmed milk powder was used to remove the non-specific background for 2 h. Afterward, anti-STAT3 monoclonal antibody (1:2,000, #9139S, Cell Signaling Technology, USA), IL-6Rα antibody (1:1,000, # sc-373708, Santa Cruz Biotechnology, USA) was added and incubated overnight at 4°C. On the next day, the PBST membrane was cleaned and incubated with HRP labeled 1purl 2000 sheep anti-rabbit second antibody for 30 min. The Supersignal West Femto/PicoHRP sensitive chemiluminescence substrate was selected to develop the nitrocellulose membrane and β-actin (1:5,000, #A5441, Sigma, USA) was used as a control. The above experiments were repeated at least three times, and the odyssey machine scanned the film as well as collected the image. The Image-J software analyzes the gray value of the target strip.

### Eyeball and Retina Detection

The retina was obtained by the anatomical microscope (Leica LAS EZ, Germany). Five hundred microliters of lectin GS-IB4 (Invitrogen, USA) was added to the retina to mark retinal vessels. The retina was flattened through four incisions and placed on a glass slide. The composition of the ischemic retinal region, the number of branches of ducts, and the clock time of the neovascularization plexus were compared with Adobe Photoshop CS6 and ImageJ software.

### Rat Retinal Cells Acquisition and Processing

The retinal tissue was cut into pieces, digested with 25 g/L trypsin lmL, centrifuged with 1,000 r/min (centrifugal radius 3 cm) for 5 min supernatant was discarded. The cell suspension was prepared by blowing an 8 mL cell culture medium and then inoculated into a petri dish. When the cells were fused into monolayer cells, they were gently blown and moved to the centrifuge tube. 1000 r/min (centrifugal radius 3 cm) was centrifuged for 5 min, and the supernatant was discarded. Added DMEM/F12 culture medium containing fetal bovine serum, carefully blow well, added an equal amount to two 35 mm Petri dishes. Afterward, added 1 ml of DMEM/F12 culture medium containing 100 g/L fetal bovine serum to each petri dish and put it into the incubator. Embryonic retinal vascular endothelial cells were grouped and were added to the complete culture medium and then treated with 250 μmol/L CoCl_2_ (#C8661-100G, Sigma, USA) for 72 h.

### Histological Analysis and Immunofluorescent Staining

For the histological analysis, the retinal tissues were dehydrated, embedded in paraffin wax, and serially sectioned. The sections were de-waxed in xylene, rehydrated, and stained with HE. The sections were photographed using a fluorescent microscope (Zeiss Axio Imager2, Germany). The sections were finally photographed by the Zeiss microscope.

### Data Analysis and Statistics

Data were expressed as the mean ±standard deviation (SD) and analyzed by SPSS V16.0 (SPSSInc., USA) software. The mean value between two groups was compared by Student's two-tailed *t*-test. ^*^P < 0.05 was considered statistically significant.

## Results

### The Observation of Retinal Vessels Using HE Stains and Immunofluorescence

**Figure 1:**
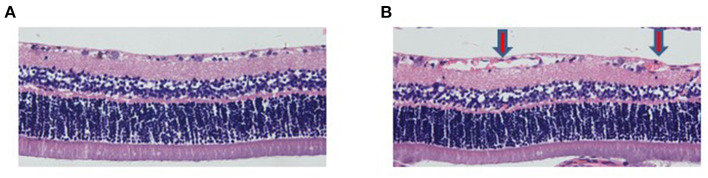
The number of retinal vessels in the hyperoxia group increased. HE staining showed retinal vessels in the air group **(A)** and hyperoxia group **(B)** (200X). The red arrow represents the retinal hyperplastic capillary plexus.

**Figure 2:**
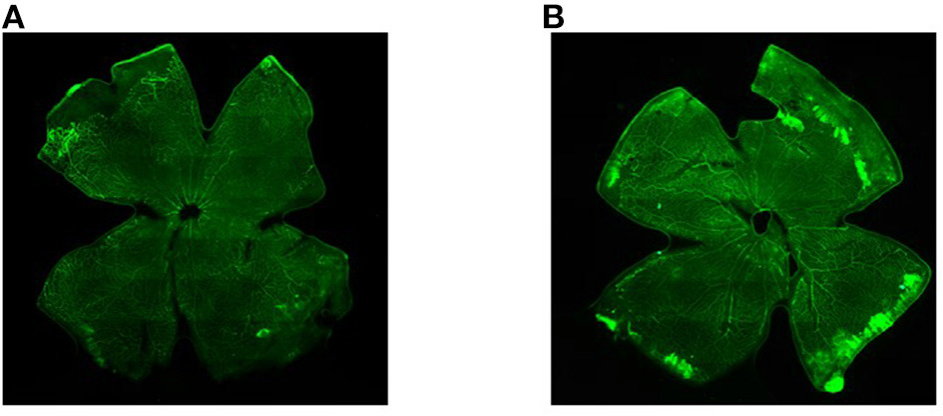
The retinal vessels in the air group and the hyperoxia group under the fluorescence microscope. **(A)** Air group, and **(B)** hyperoxia group.

The results showed that the number of retinal vessels in the hyperoxia group was significantly higher than that in the air group (Figure 1, *P* < 0.05). In the hyperoxia group, the retinal vessels were tortuous. In some regional blood vessels, they were both tortuous and dilated. However, leakage from the vascular fluorescence group was more serious, which were shown in Figure 2.

### The Expression of STAT3, IL-6R, and HAMP mRNA

**Figure 3:**
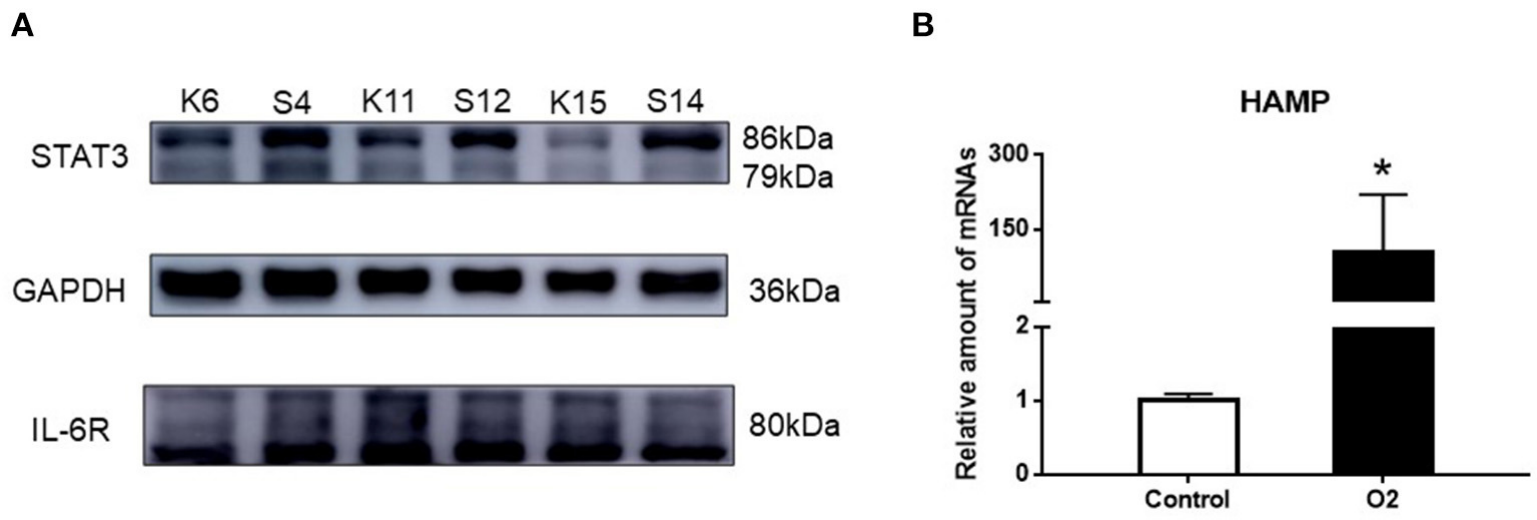
The expression of STAT3, IL-6R, and HAMP mRNA. **(A)** Western blot result showing the expression of STAT3 and IL-6R in the hyperoxia and air groups. **(B)** PCR result showing the expression of HAMP mRNA in the hyperoxia and air groups. Air group: K6, K11, K15; Hyperoxia group: S4, S12, S14 (*p < 0.05).

There was a significant increase in the expression of STAT3 protein after stimulating hyperoxia. However, IL-6R expression was similar in the two groups. Additionally, the expression of HAMP in the hyperoxia group was significantly higher than that in the air group (Figure 3, *P*< *0.05*).

### The Expression of STAT3 in Retinal Cells and VEGF mRNA

**Figure 4:**
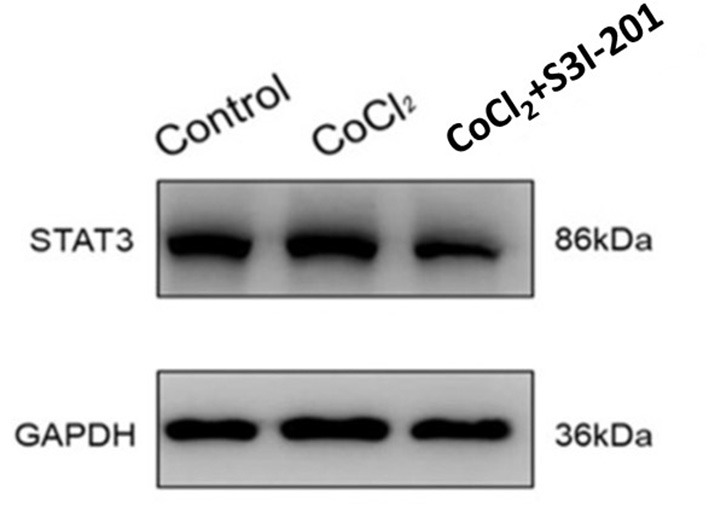
Western blot showing the expression of STAT3 in retinal cells treated with 250 μmol/L CoCl_2_ and S3I-201.

**Figure 5:**
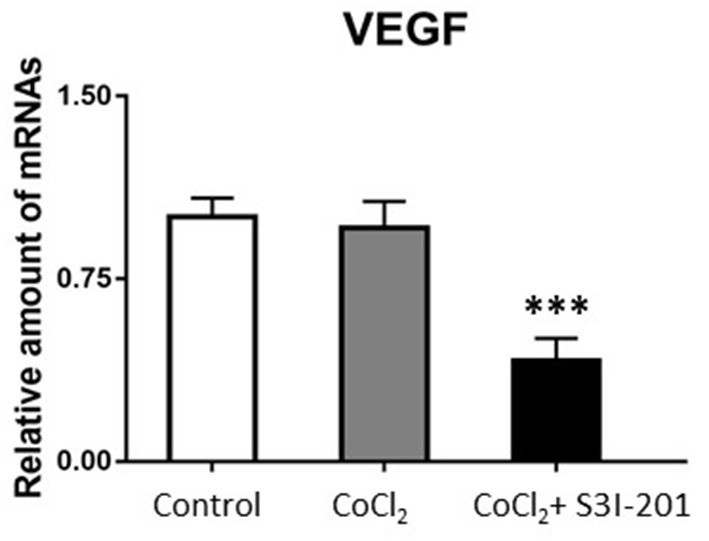
PCR result showed VEGF mRNA expression in retinal cells treated with 250 μmol/L CoCl_2_ and S3I-201 (***p < 0.05).

After 250 μmol/L CoCl_2_ treatment, the expression of STAT3 increased, whereas during treatment with S3I-201, its expression decreased (Figure 4). Additionally, after treatment with 250 μmol/L CoCl_2_ for 72 h, the expression of VEGF mRNA in retinal cells decreased, and the addition of S3I-201 also decreased its expression (Figure 5).

Taken together, our data showed that the activation of STAT3 increased the expression of hepcidin protein, thereby showing the role in ROP treatment. In addition, the S3I-201 inhibitor could inhibit the expression of STAT3 and VEGF mRNA, potentially preventing ROP.

## Discussion

In this study, we showed that the expression of STAT3 protein and HAMP mRNA increased significantly after hyperoxia stimulation or treated by cobalt chloride, indicating the potential role of STAT3 signal pathway activation and hepcidin expression in the pathogenesis of ROP. And the treatment of STAT3 pathway inhibitor S3I-201 decreased STAT3 and VEGF mRNA expression, showing that when there is a mutation or blockage on the STAT3 gene, VEGF expression will decrease accordingly.

It should be noted that excess iron caused by multiple blood transfusions and STAT3 signal transduction pathway activation play essential roles in the occurrence and development of ROP (23). And VEGF expression decreases accordingly when the STAT3 gene is blocked (24). A study by Leske et al. (25) revealed that VEGF mRNA expression in the retinopathy group of premature mice was significantly increased when neovascularization was maximized. In rats, the early stage of ROP shows that hyperoxia inhibits VEGF mRNA expression, reduces VEGF production, inhibits the development of normal retinal vessels, and degenerates the original vessels. This causes apoptosis of endothelial cells and leads to vascular occlusion (26–28). During the late stage of ROP, the relative hypoxia of the retina is aggravated, and a significant increase in VEGF expression in the retina occurs, thereby promoting the formation and proliferation of pathological neovascularization. In this study, the expression of STAT3 in retinal cells treated with cobalt chloride increased, but VEGF mRNA expression did not increase. The reason could be that VEGF is regulated by multiple signal transduction pathways and is timely-related to the pathological stage of ROP.

Hepcidin, regulated by the JAK/STAT3 signal pathway, is a 25 amino acids antimicrobial polypeptide transcripted by HAMP and secreted into blood circulation by the liver. It can regulate iron absorption in the intestines and its release in macrophages. It also plays a negative role in the absorption of iron in the intestinal tract and reduces excess iron in the body (29). Another study indicated that the binding of the STAT protein to the receptor is phosphorylated by kinase JAK (30). The activated STAT protein enters the nucleus in the form of a dimer binding to the target gene, regulates gene transcription, and induces expression of various downstream target genes related to biological behaviors such as cell differentiation, apoptosis, and angiogenesis (31). Nonetheless, in the hypoxic human retinal microvascular endothelial cells (HRMECs) model induced by cobalt chloride, we found that the expression of STAT3 and HAMP mRNA increased. In contrast, the expression of STAT3 decreased after adding an S3I-201 inhibitor. This confirmed the biological function of hepcidin and S3I-201, an inhibitor of the STAT3 pathway, and further clarified that the STAT3 signal pathway positively regulates the expression of hepcidin during the pathogenesis of ROP.

A highlight of this study is that when the iron is in abundance, iron modulin increases, and the iron absorption may be barred by the loss of the function of the iron transport membrane transporter. Hence, the iron in the blood decreases, and the iron balance is reached. Therefore, we conclude that the increased expression of STAT3 induced by hyperoxia can be compensatory for the body. This aims to alleviate further the phenomenon of iron overload caused by hyperoxia. Thus, playing a role of self-protection and avoiding the further aggravation of ROP.

Given the findings from the present study, iron-regulating pathway might be considered a potential vascular-targeting, anti-proliferation treatment strategy for retinopathy. These findings may contributing to a new generation of targeted therapeutic drugs to treat and prevent ROP. However, its specific mechanism still requires further studies.

## Data Availability Statement

The raw data supporting the conclusions of this article will be made available by the authors, without undue reservation.

## Ethics Statement

The animal study was reviewed and approved by Ethics Committee of Guangdong Women and Children's Hospital.

## Author Contributions

CN, JJ, and JR were responsible for experimental design and data interpretation. JR, WO, XL, JX, GL, SH, and LH conducted experiments. HL and JG designed the computational framework and analyzed the data. JR, HL, and CN wrote the manuscript, with editing and revising assistance from JJ. All authors discussed the results and contributed to the final manuscript.

## Funding

This work was funded by the National Natural Science Foundation of Guangdong (2018A030313605), Guangdong Medical Science and Technology Research Foundation (A2020276), Natural Science Foundation of Shenzhen City (JCYJ20190809173419560), Science and Technology Program of Guangzhou, China (201804010090 and 201804010363), and National Natural Science Foundation of Guangdong (2019A1515011417).

## Conflict of Interest

The authors declare that the research was conducted in the absence of any commercial or financial relationships that could be construed as a potential conflict of interest.

## Publisher's Note

All claims expressed in this article are solely those of the authors and do not necessarily represent those of their affiliated organizations, or those of the publisher, the editors and the reviewers. Any product that may be evaluated in this article, or claim that may be made by its manufacturer, is not guaranteed or endorsed by the publisher.

## References

[1] Bashinsky Alice L. Retinopathy of prematurity. N Carolina Med J. (2017) 78:124–8. 10.18043/ncm.78.2.124 28420777

[2] Dogra MR Katoch D Dogra M. An update on retinopathy of prematurity (ROP). Indian J Pediatr. (2017) 84:930–6. 10.1007/s12098-017-2404-3 28674824

[3] Multi center investigation and cooperation group on retinopathy of prematurity. Multicenter survey on the clinical features and fundus lesions of retinopathy in premature infants in mainland China. Chin J Evid Based Pediatr. (2015) 10:161–5. 10.3969/j.issn.1673-5501.2015.03.001

[4] Jagła M Peterko A Olesińska K Szymońska I Kwinta P. Prediction of severe retinopathy of prematurity using the WINROP algorithm in a cohort from Malopolska. A retrospective, single-center study. Dev Period Med. (2017) 21:336–43. 29291361 10.34763/devperiodmed.20172104.336343 PMC8522935

[5] Janet E Madden Deborah L Bobola. A data-driven approach to retinopathy of prematurity prevention leads to dramatic change. Adv Neonatal Care. (2010) 10:182–7. 10.1097/ANC.0b013e3181e94176 20697215

[6] Hartnett ME. Pathophysiology and mechanisms of severe retinopathy of prematurity. Ophthalmology. (2015) 122:200–10. 10.1016/j.ophtha.2014.07.050 25444347 PMC4277936

[7] Smith LE Hard AL Hellström A. The biology of retinopathy of prematurity: how knowledge of pathogenesis guides treatment. Clin Perinatol. (2013) 40:201–14. 10.1016/j.clp.2013.02.002 23719305 PMC3673697

[8] Alryalat SA AI Oweidat K AI-Amer A Khader A Ajaj A Alessa Z. Perinatal events predicting retinopathy of prematurity in extremely pre-trem infants. J Neonatal Perinatal Med. (2020) 13:261–6. 10.3233/NPM-190336 32250325

[9] Stefano N Gnocchini F Pantanetti M Battistini P Carnielli VP. The importance of oxygen control reaffirmed: experience of ROP reduction at a single tertiary care center. J Pediatr Ophthalmol Strabismus. (2014) 51:112–5. 10.3928/01913913-20140220-05 24804305

[10] Kabataş EU Kurtul BE Altiaylik Özer P Kabatas N. Comparison of intravitreal bevacizumab, intravitreal ranibizumab and laser photocoagulation for treatment of type 1 retinopathy of prematurity in Turkish preterm children. Curr Eye Res. (2017) 42:1054–8. 10.1080/02713683.2016.1264607 28128986

[11] Beharry KD Valencia GB Lazzaro DR Aranda JV. Pharmacologic interventions for the prevention and treatment of retinopathy of prematurity. Semin Perinatol. (2016) 40:189–202. 10.1053/j.semperi.2015.12.006 26831641 PMC4808450

[12] Fang JL Sorita A Carey WA Colby CE Murad MH Alahdab F. Interventions to prevent retinopathy of prematurity: a meta-analysis. Pediatrics. (2016) 137:e20153387. 10.1542/peds.2015-3387 26962240

[13] Eftekhari Milani A Hassanpoor N Mousavi Mirkala M Taheri A Golizade A Niyousha MR. Intravitreal bevacizumab injection in aggressive posterior retinopathy of prematurity compared with type I retinopathy of prematurity. Int Ophthalmol. (2020) 40:477–82. 10.1007/s10792-019-01208-3 31712928

[14] Da Silva AR Neves J Mleczko-Sanecka K Tandon A Sauer SW Hentze MW. Cellular citrate levels establish a regulatory link between energy metabolism and the hepatic iron hormone hepcidin. J Mol Med. (2017) 95:851–60. 10.1007/s00109-017-1551-3 28585096

[15] Stoian I Manolescu B Atanasiu V Lupescu O Busu C. IL-6 - STAT-3 - hepcidin: linking inflammation to the iron metabolism. Rom J Intern Med. (2007) 45:305–9. 18333366

[16] He Q Zhang H Du XZ Guo TN Ye FQ. The design, synthesis and biological activity of small molecule inhibitors targeting STAT3 signaling pathway. J Wezhou Med Univ. (2019) 49:333–8. 10.3969/j.issn.2095-9400.2019.05.004

[17] Gao DD Bao KT Zhang MM Li YX. Design, synthesis and biological evaluation of small-molecule inhibitors of signal transducer and activator of transcription 3 (STAT3) signaling pathway. Chin J Organ Chem. (2016) 36:1854–62. 10.6023/cjoc20160203027727126

[18] Cheng WX Huang H Chen JH Zhang TT Zhu GY Zheng ZT. Genistein inhibits angiogenesis developed during rheumatoid arthritis through the IL-6/JAK2/STAT3/VEGF signalling pathway. J Orthop Translat. (2020) 22:92–100. 10.1016/j.jot.2019.07.007 32440504 PMC7231959

[19] Cui YF Li PS Hao SF. Study on expression levels of STAT3, MMPs, and VEGF in patients with endometriosis. Matern Child Health Care China. (2019) 34:250–2. 10.7620/zgfybj.j.issn.1001-4411.2019.02.04

[20] Tan YN Tao YG Li LL Liu SF Gao Y. Regulation of human VEGF by expression latent membrane protein 1 encoded by EB virus *via* STAT3 in nasopharyngeal carcinoma cell line. Prog Biochem Biophys. (2004) 5:427–31. 10.3321/j.issn:1000-3282.2004.05.008

[21] Devi SU Ramasarma T. The iron-binding properties of fermodulin. Indian J Biochem Biophys. (1987) 24:119–23. 3428908

[22] Menon AS Devi SU Ramasarma T. Purification and characterization of fermodulin, an Fe2+-dependent inhibitor protein of 3-hydroxy-3-methylglutaryl-CoA reductase. Arch Biochem Biophys. (1985) 239:342–51. 10.1016/0003-9861(85)90697-6 4004269

[23] Stutchfield CJ Jain A Odd D Williams C Markham R. Foetal haemoglobin, blood transfusion, and retinopathy of prematurity in very preterm infants: a pilot prospective cohort study. Eye (Lond). (2017) 31:1451–5. 10.1038/eye.2017.76 28548651 PMC5639193

[24] Niu G Wright KL Huang M Song L Haura E Turkson J. Constitutive Stat3 activity up-regulates VEGF expression and tumor angiogenesis. Oncogene. (2002) 21:2000–8. 10.1038/sj.onc.1205260 11960372

[25] Leske DA Wu JM Mookadam M. The relationship of retinal VEGF and retinal IGF-lmRNA with neovascularization in an acidosis-induced model of retinopathy of prematurity. Curr Eye Res. (2006) 31:163–9. 10.1080/02713680500507281 16500767

[26] Chen J Smith LEH. Retinopathy of prematurity. Angiogenesis. (2007) 10:133–40. 10.1007/s10456-007-9066-0 17332988

[27] Penn JS Madan A Caldwell RB Bartoli M Caldwell RW Hartnett ME. Vascular endothelial growth factor in eye disease. Prog Retinal Eye Res. (2008) 27:331–71. 10.1016/j.preteyeres.2008.05.001 18653375 PMC3682685

[28] Byfield G Budd S Hartnett ME. The role of supplemental oxygen and JAK/STAT signaling in intravitreous neovascularization in a ROP rat model. Invest Ophthalmol Vis Sci. (2009) 50:3360–5. 10.1167/iovs.08-3256 19264880 PMC3682836

[29] Liu N Zhang WF Zhang BX Wu XL Ma FT Zhao XQ. Research progress on the relationship between iron overload related diseases and hepcidin. Guangdong Med J. (2016) 37:2048–9.

[30] Kiu H Nicholson SE. Biology and significance of the JAK/STAT signalling pathways. Growth Factors (Chur, Switzerland). (2012) 30:88–106. 10.3109/08977194.2012.660936 22339650 PMC3762697

[31] Gu L Zhang LJ Liu LF Hu D Liang WT. Inhibitors of JAK/ STAT signaling pathway and lung cance. J Mod Oncol. (2018) 26:314–7. 10.3969/j.issn.1672-4992.2018.02.039

